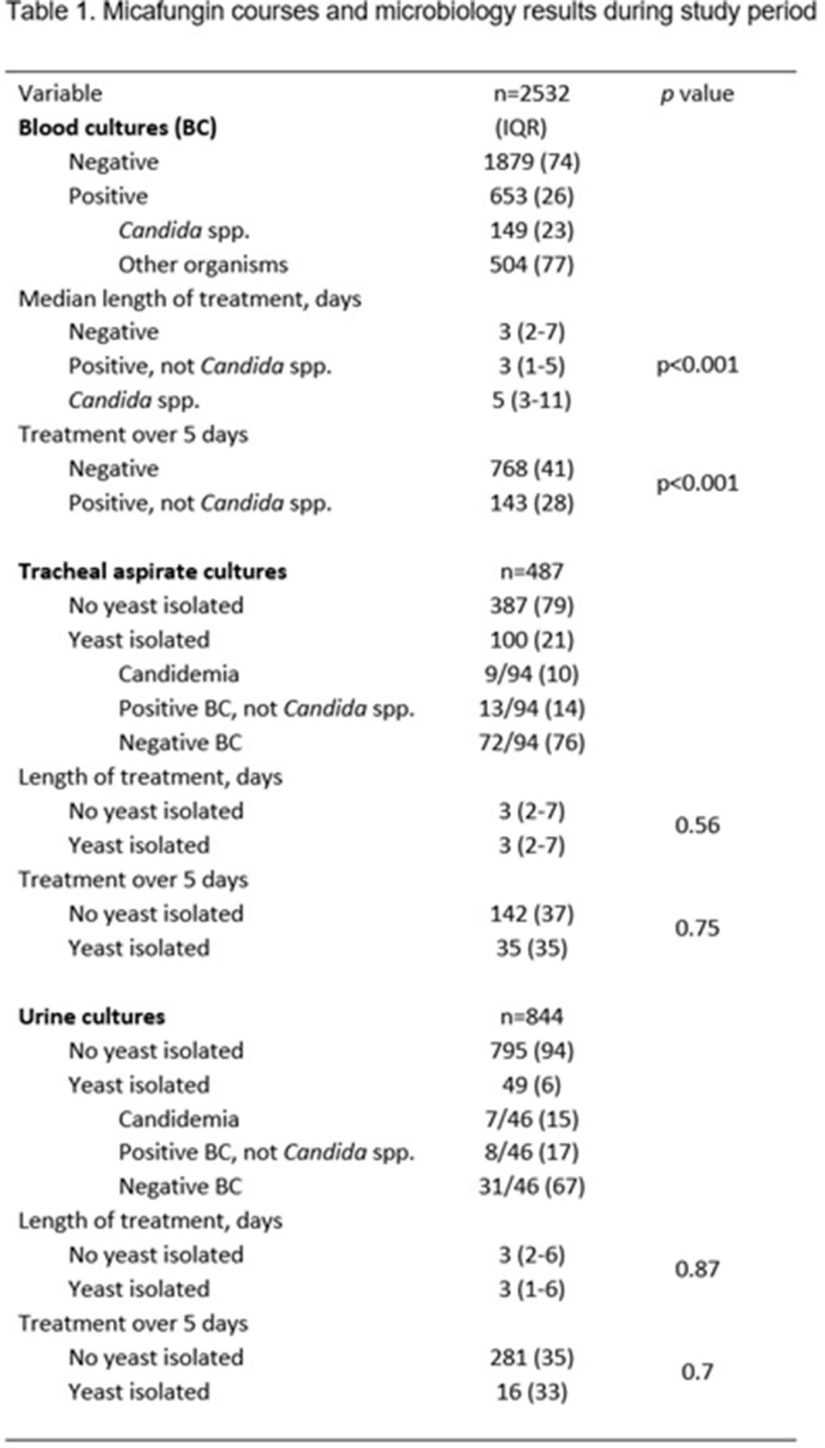# Identification of Potentially Unnecessary Micafungin Use Patterns: Opportunities for Antifungal Stewardship Interventions

**DOI:** 10.1017/ash.2021.60

**Published:** 2021-07-29

**Authors:** Miguel Chavez Concha, Kevin Hsueh, Michael Durkin, Andrej Spec

## Abstract

**Background:** Echinocandins are used as first-line therapy for suspected and confirmed *Candida* spp, and its indiscriminate use may drive selection for echinocandin resistance. We evaluated patterns of use of micafungin to identify opportunities for antifungal stewardship. **Methods:** We identified all micafungin completed orders and microbiological test result data from July 2018 to November 2020 among hospitalized patients in Barnes-Jewish Hospital. Continuous micafungin courses with <48 hours of interruption were considered independent courses. We evaluated micafungin use in 3 scenarios in which its use may be unnecessary: (1) patients with blood cultures negative for *Candida* spp, (2) patients with recovery of yeast or *Candida* spp from tracheal aspirates, and (3) patients with recovery of yeast or *Candida* spp from urine cultures. We only included micafungin courses if they were initiated within 5 days of blood culture collection or up to 4 days after tracheal or urine culture collection to account for incubation and decision to initiate treatment. **Results:** We found 3,381 micafungin courses in 3,287 admissions. Of these, 2,532 courses had blood culture collection around micafungin initiation and were included in the first analysis: 1,879 (74%) were negative, 149 (6%) had *Candida* spp isolated in the blood, and 504 (20%) had positive blood cultures for other organisms. Micafungin was given for a median duration of 3 days (IQR, 2–7) to those with negative blood cultures and for 3 days (IQR, 1–5) to those with positive blood cultures without candidemia (p < 0.001), and prolonged durations of more than 5 days was seen in 768/1879 (41%) and 143/504 (28%) of courses, respectively (p <0.001). A total of 487 micafungin courses were initiated after tracheal aspirate culture collection. Those with yeast isolated (n = 100, 21%) received similar micafungin duration compared to those that had no yeast isolated [3 (2-7 IQR) vs. 3 (2-7) days, respectively; p = 0.56). Finally, a total of 844 micafungin courses started after urine culture collection. A total of 49 (6%) had yeast isolated from the urine and treatment duration was similar to those that did not [3 (1-6 IQR) vs. 3 (2-6) days, respectively; p = 0.87). **Conclusions:** Echinocandin treatment courses did not differ when a yeast was identified from a tracheal isolate or urine specimen. However, a substantial proportion of treatment courses were prolonged in those with negative *Candida* spp in the blood, suggesting opportunities for antifungal stewardship interventions.

**Funding:** No

**Disclosures:** None

**Table 1.**